# Supplementary material for: Hot spot prediction in protein-protein interactions by an ensemble system
Source: BMC Syst Biol. 2018 Dec 31;12(Suppl 9):132. doi: 10.1186/s12918-018-0665-8 (PMC6311905; doi:10.1186/s12918-018-0665-8)
Supplement: Supplementary file 2 — All relevant MATLAB code. A simple Matlab implement of our predictor. (ZIP 19,742 kb) [file 12918_2018_665_MOESM2_ESM.zip › Additional file 2/Read ASA/ASA website/SKEMPI/2J1KC.htm]

 NetSurfP - Protein Surface Accessibility and Secondary Structure Predictions

  

|  |  |  |
| --- | --- | --- |
|  |  | NetSurfP - Protein Surface Accessibility and Secondary Structure Predictions  Technical University of Denmark |

  


---

```
# For publication of results, please cite:
# A generic method for assignment of reliability scores applied to solvent accessibility predictions.
# Bent Petersen, Thomas Nordahl Petersen, Pernille Andersen, Morten Nielsen and Claus Lundegaard
# BMC Structural Biology 2009, 9:51 doi:10.1186/1472-6807-9-51
#
# Column 1: Class assignment - B for buried or E for Exposed - Threshold: 25% exposure, but not based on RSA
# Column 2: Amino acid
# Column 3: Sequence name
# Column 4: Amino acid number
# Column 5: Relative Surface Accessibility - RSA
# Column 6: Absolute Surface Accessibility
# Column 7: Z-fit score for RSA prediction
# Column 8: Probability for Alpha-Helix
# Column 9: Probability for Beta-strand
# Column 10: Probability for Coil
E A  2J1KC                  1    0.739  81.493  -0.562   0.000   0.000   0.999
E P  2J1KC                  2    0.468  66.452  -0.535   0.005   0.015   0.979
B I  2J1KC                  3    0.204  37.759  -0.397   0.020   0.205   0.775
B T  2J1KC                  4    0.140  19.487  -0.160   0.021   0.279   0.699
B L  2J1KC                  5    0.085  15.490  -0.142   0.021   0.451   0.528
B W  2J1KC                  6    0.088  21.140   0.067   0.022   0.552   0.426
B T  2J1KC                  7    0.114  15.867  -0.312   0.021   0.451   0.528
B G  2J1KC                  8    0.221  17.361  -0.109   0.004   0.197   0.799
E P  2J1KC                  9    0.376  53.383  -1.040   0.005   0.045   0.951
E G  2J1KC                 10    0.506  39.799  -1.000   0.005   0.015   0.979
B P  2J1KC                 11    0.298  42.258  -0.826   0.018   0.019   0.964
E S  2J1KC                 12    0.422  49.458  -1.213   0.018   0.019   0.964
B I  2J1KC                 13    0.266  49.173  -1.060   0.018   0.047   0.935
E N  2J1KC                 14    0.345  50.479  -0.923   0.004   0.138   0.858
B G  2J1KC                 15    0.044   3.463  -0.246   0.004   0.420   0.576
E F  2J1KC                 16    0.409  82.126   1.514   0.002   0.816   0.182
B I  2J1KC                 17    0.119  21.941   0.191   0.001   0.900   0.099
E N  2J1KC                 18    0.472  69.145  -0.070   0.002   0.816   0.182
E D  2J1KC                 19    0.538  77.526  -0.624   0.004   0.514   0.481
E T  2J1KC                 20    0.498  69.017  -0.356   0.004   0.138   0.858
E P  2J1KC                 21    0.495  70.311  -0.408   0.005   0.045   0.951
E V  2J1KC                 22    0.468  71.993  -0.093   0.005   0.045   0.951
B I  2J1KC                 23    0.044   8.066  -0.357   0.005   0.262   0.733
E R  2J1KC                 24    0.261  59.655   1.610   0.003   0.718   0.279
B C  2J1KC                 25    0.028   3.875   0.868   0.001   0.959   0.040
B F  2J1KC                 26    0.049   9.814   0.755   0.001   0.959   0.040
B I  2J1KC                 27    0.017   3.145   1.441   0.001   0.959   0.040
B C  2J1KC                 28    0.027   3.805   0.581   0.001   0.959   0.040
B L  2J1KC                 29    0.019   3.534   1.214   0.001   0.959   0.040
B T  2J1KC                 30    0.045   6.311  -0.105   0.001   0.900   0.099
B R  2J1KC                 31    0.239  54.708   0.297   0.004   0.616   0.381
B D  2J1KC                 32    0.191  27.538  -1.161   0.004   0.138   0.858
E S  2J1KC                 33    0.362  42.450  -1.321   0.005   0.015   0.979
B N  2J1KC                 34    0.206  30.100  -2.502   0.004   0.197   0.799
B L  2J1KC                 35    0.203  37.243   0.749   0.002   0.816   0.182
B V  2J1KC                 36    0.065  10.037  -0.117   0.001   0.959   0.040
B T  2J1KC                 37    0.134  18.614   0.427   0.001   0.959   0.040
B V  2J1KC                 38    0.048   7.393  -0.528   0.001   0.959   0.040
B N  2J1KC                 39    0.126  18.417  -0.097   0.001   0.959   0.040
B A  2J1KC                 40    0.040   4.364   0.135   0.001   0.959   0.040
B S  2J1KC                 41    0.094  11.064   0.034   0.000   0.983   0.017
B F  2J1KC                 42    0.064  12.744   0.092   0.000   0.983   0.017
B V  2J1KC                 43    0.145  22.348   0.030   0.001   0.959   0.040
B G  2J1KC                 44    0.122   9.594  -1.370   0.001   0.900   0.099
E E  2J1KC                 45    0.423  73.811   0.407   0.004   0.616   0.381
B G  2J1KC                 46    0.317  24.972  -1.474   0.005   0.336   0.660
E G  2J1KC                 47    0.590  46.417  -0.537   0.004   0.138   0.858
E Y  2J1KC                 48    0.283  60.520  -0.369   0.005   0.336   0.660
E R  2J1KC                 49    0.504 115.302  -0.198   0.004   0.616   0.381
E I  2J1KC                 50    0.424  78.514  -0.747   0.023   0.655   0.322
B V  2J1KC                 51    0.182  28.020  -1.021   0.021   0.451   0.528
E S  2J1KC                 52    0.434  50.841  -0.737   0.004   0.197   0.799
E P  2J1KC                 53    0.467  66.310  -1.220   0.004   0.085   0.910
E T  2J1KC                 54    0.489  67.894  -0.816   0.004   0.085   0.910
E Q  2J1KC                 55    0.553  98.766  -0.332   0.005   0.336   0.660
E S  2J1KC                 56    0.488  57.170   0.193   0.004   0.616   0.381
E Q  2J1KC                 57    0.369  65.868   0.447   0.001   0.900   0.099
B F  2J1KC                 58    0.077  15.354   0.552   0.001   0.959   0.040
E S  2J1KC                 59    0.294  34.504   1.014   0.001   0.959   0.040
B L  2J1KC                 60    0.029   5.347   0.660   0.000   0.983   0.017
B I  2J1KC                 61    0.216  39.979   1.165   0.000   0.983   0.017
B M  2J1KC                 62    0.040   8.084   0.631   0.001   0.959   0.040
B E  2J1KC                 63    0.187  32.651   1.076   0.001   0.959   0.040
B F  2J1KC                 64    0.044   8.730   0.133   0.002   0.816   0.182
B D  2J1KC                 65    0.229  33.013   0.301   0.019   0.141   0.840
E Q  2J1KC                 66    0.617 110.268  -1.128   0.018   0.047   0.935
E F  2J1KC                 67    0.538 107.936  -0.095   0.018   0.047   0.935
B G  2J1KC                 68    0.148  11.679  -1.369   0.018   0.088   0.893
E Q  2J1KC                 69    0.374  66.725   0.564   0.021   0.451   0.528
B L  2J1KC                 70    0.127  23.345  -0.413   0.023   0.655   0.322
B M  2J1KC                 71    0.216  43.162   0.361   0.022   0.552   0.426
E S  2J1KC                 72    0.548  64.249  -1.222   0.021   0.451   0.528
E T  2J1KC                 73    0.437  60.584  -1.175   0.020   0.205   0.775
E G  2J1KC                 74    0.380  29.882  -2.264   0.019   0.141   0.840
E N  2J1KC                 75    0.405  59.263  -1.879   0.020   0.205   0.775
B I  2J1KC                 76    0.195  36.093  -1.233   0.020   0.205   0.775
E N  2J1KC                 77    0.449  65.704  -0.336   0.020   0.205   0.775
E S  2J1KC                 78    0.456  53.490  -1.181   0.021   0.279   0.699
E T  2J1KC                 79    0.561  77.880  -1.155   0.022   0.359   0.619
B T  2J1KC                 80    0.213  29.612  -0.633   0.022   0.552   0.426
E T  2J1KC                 81    0.331  45.965  -0.938   0.023   0.655   0.322
B W  2J1KC                 82    0.182  43.747  -0.961   0.022   0.552   0.426
E G  2J1KC                 83    0.310  24.421  -1.965   0.021   0.451   0.528
E E  2J1KC                 84    0.302  52.742  -1.100   0.022   0.552   0.426
E K  2J1KC                 85    0.375  77.137  -0.908   0.021   0.451   0.528
E P  2J1KC                 86    0.343  48.615  -1.844   0.021   0.279   0.699
B W  2J1KC                 87    0.250  60.029  -1.827   0.019   0.141   0.840
E G  2J1KC                 88    0.519  40.806  -1.383   0.018   0.088   0.893
B N  2J1KC                 89    0.270  39.513  -1.864   0.052   0.084   0.864
E N  2J1KC                 90    0.415  60.785  -1.639   0.052   0.084   0.864
E T  2J1KC                 91    0.406  56.271  -2.054   0.052   0.084   0.864
E V  2J1KC                 92    0.412  63.355  -2.359   0.053   0.043   0.903
E Q  2J1KC                 93    0.397  70.886  -1.545   0.053   0.043   0.903
B P  2J1KC                 94    0.295  41.903  -2.209   0.115   0.016   0.868
E R  2J1KC                 95    0.365  83.699  -1.060   0.113   0.043   0.844
B P  2J1KC                 96    0.223  31.615  -0.880   0.268   0.043   0.689
B S  2J1KC                 97    0.243  28.444  -1.973   0.268   0.043   0.689
E H  2J1KC                 98    0.324  58.899  -1.091   0.354   0.048   0.598
B T  2J1KC                 99    0.239  33.191  -0.724   0.354   0.048   0.598
B W  2J1KC                100    0.214  51.443  -0.678   0.455   0.046   0.498
E K  2J1KC                101    0.361  74.258  -0.217   0.354   0.048   0.598
B L  2J1KC                102    0.279  51.103  -0.932   0.268   0.043   0.689
B C  2J1KC                103    0.067   9.365  -1.766   0.191   0.086   0.723
B M  2J1KC                104    0.079  15.748  -0.143   0.052   0.084   0.864
B P  2J1KC                105    0.103  14.644  -0.815   0.052   0.084   0.864
B N  2J1KC                106    0.237  34.653  -1.064   0.019   0.141   0.840
B R  2J1KC                107    0.219  50.243  -0.644   0.021   0.279   0.699
E E  2J1KC                108    0.291  50.785  -0.868   0.021   0.451   0.528
B V  2J1KC                109    0.082  12.557  -1.110   0.021   0.451   0.528
B Y  2J1KC                110    0.156  33.380   0.511   0.021   0.451   0.528
B S  2J1KC                111    0.158  18.506  -1.609   0.021   0.279   0.699
E T  2J1KC                112    0.372  51.541   0.794   0.019   0.141   0.840
E P  2J1KC                113    0.365  51.822  -1.297   0.018   0.088   0.893
E A  2J1KC                114    0.572  63.067  -0.853   0.018   0.047   0.935
E A  2J1KC                115    0.644  70.958  -0.686   0.018   0.088   0.893
E T  2J1KC                116    0.529  73.386  -1.094   0.018   0.088   0.893
E I  2J1KC                117    0.411  75.961  -0.370   0.019   0.141   0.840
E S  2J1KC                118    0.575  67.378  -0.607   0.020   0.205   0.775
E R  2J1KC                119    0.446 102.248   0.214   0.021   0.451   0.528
B C  2J1KC                120    0.298  41.825  -0.566   0.023   0.655   0.322
B G  2J1KC                121    0.155  12.167  -0.004   0.018   0.846   0.136
B L  2J1KC                122    0.191  34.954   0.171   0.011   0.918   0.071
B D  2J1KC                123    0.102  14.713  -0.767   0.011   0.918   0.071
B S  2J1KC                124    0.097  11.368  -0.277   0.011   0.918   0.071
B I  2J1KC                125    0.045   8.343   0.229   0.006   0.962   0.032
B A  2J1KC                126    0.084   9.224   0.034   0.011   0.918   0.071
B V  2J1KC                127    0.092  14.110  -0.352   0.002   0.816   0.182
E D  2J1KC                128    0.371  53.490  -0.107   0.005   0.262   0.733
E G  2J1KC                129    0.498  39.185  -1.802   0.005   0.045   0.951
E A  2J1KC                130    0.534  58.803  -1.059   0.005   0.045   0.951
E P  2J1KC                131    0.339  48.090  -0.993   0.004   0.085   0.910
E S  2J1KC                132    0.405  47.501  -1.263   0.004   0.085   0.910
E R  2J1KC                133    0.349  79.898   0.730   0.004   0.138   0.858
B S  2J1KC                134    0.171  20.053  -0.527   0.004   0.514   0.481
B I  2J1KC                135    0.044   8.214   0.624   0.001   0.900   0.099
E D  2J1KC                136    0.205  29.512   0.888   0.001   0.959   0.040
B C  2J1KC                137    0.024   3.412   0.774   0.001   0.959   0.040
B M  2J1KC                138    0.138  27.614   1.023   0.001   0.959   0.040
B L  2J1KC                139    0.030   5.456   0.339   0.001   0.959   0.040
B I  2J1KC                140    0.105  19.481  -0.447   0.001   0.959   0.040
B I  2J1KC                141    0.110  20.276  -0.362   0.002   0.816   0.182
B N  2J1KC                142    0.186  27.230  -1.053   0.022   0.359   0.619
E K  2J1KC                143    0.417  85.695  -0.728   0.004   0.197   0.799
E P  2J1KC                144    0.499  70.808  -1.446   0.004   0.197   0.799
E K  2J1KC                145    0.458  94.272  -1.131   0.004   0.085   0.910
E G  2J1KC                146    0.618  48.613  -1.957   0.005   0.045   0.951
E V  2J1KC                147    0.424  65.200  -1.300   0.004   0.085   0.910
B A  2J1KC                148    0.154  16.927  -1.126   0.005   0.262   0.733
B T  2J1KC                149    0.194  26.880  -1.129   0.004   0.616   0.381
B Y  2J1KC                150    0.103  22.032   0.148   0.002   0.816   0.182
B T  2J1KC                151    0.056   7.809  -0.585   0.001   0.900   0.099
B L  2J1KC                152    0.033   6.097   0.351   0.001   0.959   0.040
B T  2J1KC                153    0.128  17.726   0.454   0.001   0.959   0.040
B F  2J1KC                154    0.037   7.446   0.194   0.001   0.959   0.040
E R  2J1KC                155    0.263  60.296   0.855   0.001   0.959   0.040
B F  2J1KC                156    0.063  12.704   0.282   0.001   0.900   0.099
E L  2J1KC                157    0.317  57.951   0.478   0.021   0.756   0.223
B N  2J1KC                158    0.296  43.305  -1.749   0.021   0.451   0.528
B F  2J1KC                159    0.265  53.085  -0.923   0.056   0.142   0.802
E N  2J1KC                160    0.384  56.159  -1.469   0.052   0.084   0.864
E R  2J1KC                161    0.456 104.424  -0.931   0.064   0.216   0.721
E L  2J1KC                162    0.520  95.139  -0.973   0.020   0.205   0.775
E S  2J1KC                163    0.314  36.813  -0.692   0.020   0.205   0.775
E G  2J1KC                164    0.555  43.710  -0.021   0.018   0.088   0.893
E G  2J1KC                165    0.639  50.250  -0.956   0.018   0.047   0.935
E T  2J1KC                166    0.374  51.860   0.423   0.020   0.205   0.775
E L  2J1KC                167    0.529  96.823   0.392   0.021   0.451   0.528
B F  2J1KC                168    0.109  21.856  -0.142   0.022   0.552   0.426
E K  2J1KC                169    0.566 116.488  -1.017   0.022   0.359   0.619
B T  2J1KC                170    0.181  25.160  -0.368   0.020   0.205   0.775
E D  2J1KC                171    0.359  51.674  -0.744   0.004   0.138   0.858
B V  2J1KC                172    0.280  43.067  -0.898   0.005   0.336   0.660
B L  2J1KC                173    0.086  15.710  -0.117   0.003   0.718   0.279
B T  2J1KC                174    0.161  22.275  -0.670   0.002   0.816   0.182
B F  2J1KC                175    0.054  10.818   0.309   0.001   0.900   0.099
B T  2J1KC                176    0.092  12.816  -0.304   0.001   0.900   0.099
B Y  2J1KC                177    0.118  25.302   0.493   0.001   0.900   0.099
B V  2J1KC                178    0.089  13.725  -0.073   0.018   0.846   0.136
B G  2J1KC                179    0.116   9.121  -1.009   0.003   0.718   0.279
E E  2J1KC                180    0.261  45.632  -0.331   0.004   0.616   0.381
E N  2J1KC                181    0.581  85.058  -0.308   0.022   0.359   0.619
E Q  2J1KC                182    0.837 149.560   0.616   0.016   0.005   0.979

Explain the output.  Go back.
```

---
